# The Effects on Tuberculosis Treatment Adherence from Utilising Community Health Workers: A Comparison of Selected Rural and Urban Settings in Kenya

**DOI:** 10.1371/journal.pone.0088937

**Published:** 2014-02-18

**Authors:** Jane Rahedi Ong'ang'o, Christina Mwachari, Hillary Kipruto, Simon Karanja

**Affiliations:** 1 Centre for Respiratory Disease Research, Kenya Medical Research Institute (KEMRI), Nairobi, Kenya; 2 Kenya Country Office, World Health Organisation (WHO), Nairobi, Kenya; 3 College of Health Sciences, Jomo Kenyatta University of Agriculture and Technology (JKUAT), Nairobi, Kenya; Institut de Pharmacologie et de Biologie Structurale, France

## Abstract

**Introduction:**

Community Health Workers (CHWs) have been utilised for various primary health care activities in different settings especially in developing countries. Usually when utilised in well defined terms, they have a positive impact. To support Kenya's policy on engagement of CHWs for tuberculosis (TB) control, there is need to demonstrate effects of utilising them.

**Objectives:**

This study assessed TB treatment adherence among patients who utilised CHWs in management of their illness in comparison to those who did not in urban and rural settings.

**Methods:**

A retrospective cohort study was conducted in selected health facilities using standard clinical records for each TB patient registered for treatment between 2005 to 2011. Qualitative data was collected from CHWs and health care providers.

**Results:**

The study assessed 2778 tuberculosis patients and among them 1499 (54%) utilized CHWs for their TB treatment. The urban setting in comparison with the rural setting contributed 70% of patients utilising the CHWs (p<0.001). Overall treatment adherence of the cohort was 79%. Categorizing by use of CHWs, adherence among patients who had utilized CHWs was 83% versus 68% among those that had not (p<0.001). In comparison between the rural and urban settings adherence was 76% and 81.5% (p<0.001) respectively and when categorized by use of CHWs it was 73% and 90% (p<0.001) for the rural and urban set ups respectively. Utilisation of CHWs remained significant in enhancing treatment adherence in the cohort with unadjusted and adjusted ORs; OR 2.25, (95% 1.86–2.73) p<0.001 and OR 1.98 (95% 1.51–2.5) p<0.001 respectively. It was most effective in the urban set-up, OR 2.65 (95% 2.02–3.48, p<0.001) in comparison to the rural set up, OR 0.74 (95% 0.56–0.97) p = 0.032.

**Conclusion:**

Utilisation of CHWs enhanced TB treatment adherence and the best effects were in the urban set-up.

## Introduction

Community Health Workers (CHWs) have been utilised for various primary health care activities in different parts of the world, including Asia [1], [2], United States of America (USA) [3], [4] and Africa [5], [6], [7], [8], [9]. The term “Community Health Worker” encompasses a variety of health assistants who are recruited by respective ministries in-charge of health. Usually they are trained and work with communities in which they live. It is generally felt that engagement of CHWs impacts positively on human health via encouraging increased utilization of health care services and supporting preventive health programmes [10], [11]. To ensure the sustainability and effectiveness of CHWs, it has been realised that they require some form of incentive [6], [10], [12]. To emphasise this need, the rising incidence of poverty in many sub-Saharan African countries has resulted in the dying spirit of volunteerism because people have to use their time to get an earning. Thus, any engagement that would not contribute some resources to their survival may not be sustainable. When the success by CHWs is minimal, the principal reason is usually failure of the health system to provide them with the necessary support. This was demonstrated in the Phillipines where CHWs had inadequate training, insufficient logistics support, poorly motivated schemes and lack of community support resulting to below optimal contribution from them [1]. With the dramatic increase in the burden of tuberculosis (TB) related to HIV, many countries are utilising community participation in TB control [13]. Consequently, there has been a growing demand for CHWs to take on the management of other diseases, including malaria [14], [15]. Utilisation of CHWs has the potential to positively affect the health and treatment behaviour in communities where they live and work. The challenge in utilizing them is how to determine the best effective way to implement and manage this type of public health intervention [10].

Poor adherence to treatment remains a major obstacle in the global fight against TB [16]. Reasons for non-adherence are complex and multifaceted involving more than the patients' personal characteristics and attitudes [17]. Factors, such as the chronic nature of the disease, the socio-cultural context and poverty, and interacting with physicians, nurses, and other health care workers, all affect access to and adherence to treatment [18], [19], [20], [21], [22], [23], [24]. In Kenya, the National TB Programme (NTP) provides free TB treatment that consists of a standard 6 month regimen (2 months intensive phase of combined rifampicin, isoniazid, pyrazinamide, and ethambutol (RHZE), followed by 4 months continuation phase of combined isoniazid and rifampicin (RH)) for the new TB patients diagnosed and sensitive to first line treatment. Retreatment TB patients are treated with a standard 8-month regimen (2 months intensive phase of combined streptomycin, rifampicin, isoniazid, pyrazinamide, and ethambutol (SRHZE), a continuation phase of 1 month (RHZE) followed by 5 months of combined rifampicin, isoniazid and ethambutol (RHE).

Directly observed therapy (DOT) is central to the global strategy for effective TB control launched by the WHO in 1994 and named directly observed therapy- short course [DOTS] [25]. In 2002, WHO recommended flexibility in implementing DOTS and promoted “a comprehensive and multi-factorial approach.”[26]. Qualitative studies have shown that patients who receive the support and care of their families were more likely to adhere to therapy and achieve cure [27]. Some newly patient-centered approaches have been tested in observational studies performed in Uganda, Kenya, Malawi, and Tanzania which showed that the choice of a DOT supporter by the patient, associated with the decentralization of treatment, improved treatment success rates [28], [29], [30], [31]. More studies [2], [7], [8], [32], [33] have reported good treatment outcomes when CHWs are involved in TB control.

In Kenya, most TB treatment facilities practice DOT only during clinic days, when drugs are issued weekly during the intensive phase and monthly during the continuous phase. Apart from this, each TB patient is encouraged to have a family supporter in their households to supervise DOTS. Ideally, whenever there is a defaulter of treatment, one ought to be traced by the Public Health Officers in the treatment centres but this has not been efficiently done.

In Kenya, the NTP has a policy that encourages utilisation of CHWs for DOTS supervision [34]. There have been attempts to implement this support in some of the TB treatment setups, but many a times this support ends up being unsustainable. Thus, very few treatment centres still utilise CHWs and those that do, may most probably have support from development partners. The various treatment centres that have continuously utilised CHWs for well over 5 years are more likely to be organised because of the following factors; they have well defined terms of reference describing the duties of the CHWs in the health facilities, the community and within households, there are clear guidelines with regards to the incentives provided to motivate the CHWs. In addition there is provision of supervisory support, continuing education and logistic support to enable the CHWs work. Within this whole set up there is a coordinated system that harmonises the operations of the CHWs and the professional health care providers. It is important to understand how effective the use of CHWs in the management of TB is compared to not utilizing them so that the NTP has credible information it could use to decide on engaging CHWs in TB control. This study assessed the treatment adherence of TB patients who utilised CHWs in their management in comparison to those that did not.

## Methods

### Study Design

This was a retrospective cohort study that retrieved clinical records for each TB patient from the TB treatment registers in the selected health facilities. The data collected covered a time period between the years 2005 to 2011. In addition to the quantitative data collected, qualitative data was collected from Focus group discussions (FGDs), conducted among CHWs to gather views on utilization of CHWs. In-depth interviews (IDI) were conducted among 2 health care providers who were directly involved in the supervision of CHWs. FGDs and IDI guides were used to conduct these processes.

### Study settings and population

**Figure 1:**
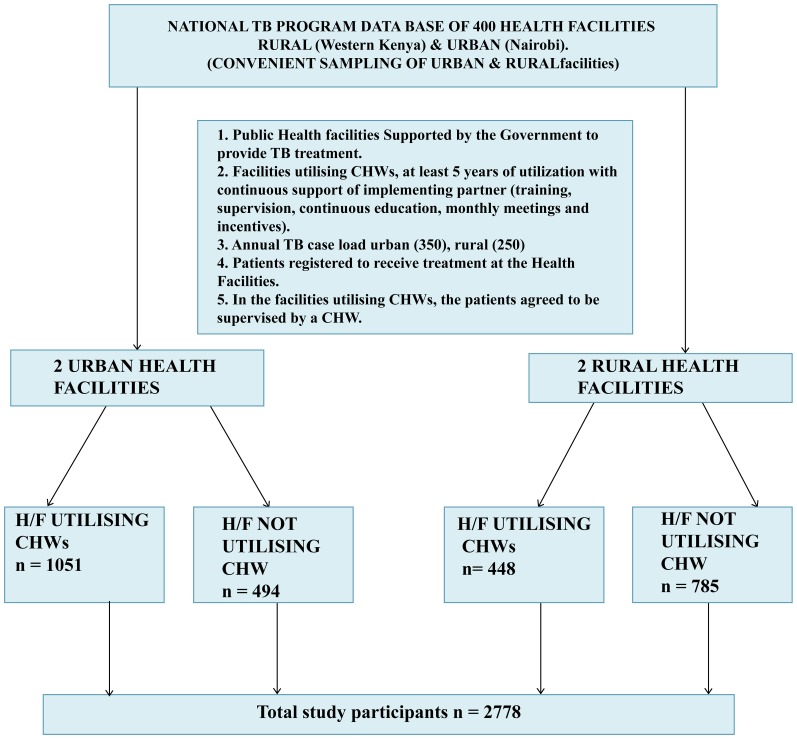
Framework of Study. H/F Health Facility.

The study was conducted in selected urban and rural settings, as guided by the criteria described in the study framework in figure 1. Two similar TB treatment health facilities were purposively selected: one engaged CHWs for TB management and the other did not. One informal settlement in Nairobi, namely, Kawangare of Dagoretti Division and Nyando District, Nyanza Province of Western Kenya represented the urban and rural set-up respectively. The Kenya national population census of 2009 categorised Kawangare within Nairobi as an urban set-up, while Nyando district was classified as a rural set-up. Within Kawangare, Riruta Health Centre was enlisted as a facility that had been utilising CHWs from the year 2003 with the support of Malteser, a development partner supporting the Ministry of Health. The support provided to the CHWs included training, supervision, continuous education, monthly meetings and incentives. The other facility that was enlisted was Wema Health Centre which had never utilized CHWs and was found within the same locality. These represented the urban setting. In the rural setting, Nyando district hospital was enlisted as the facility that had utilised CHWs from the year 2005 through the support of CDC/KEMRI who provided training, supervision, and incentive to the CHWs, while Nyabondo Mission hospital that had never utilized CHWs was included in the study as the facility for comparison. All these facilities were receiving support from the NTP for TB control. The study collected the records of all TB patients registered in the facility TB treatment registers.

### Intervention

The intervention tested in this study was the utilization of CHWs in the management of TB. The utilization of CHWs entailed the following; once a TB patient was registered to start treatment, they were attached to a trained CHW who preferably resided within the same area as the patient. Before initiation of treatment, the TB patient received personalised education from the CHW on TB treatment; risks involved in case of lack of adherence, follow-up schedule and the role of the CHW in his/her treatment. During the intensive phase of treatment, the CHW supervised DOTS at the household level once a week and in the continuous phase, once a month. The TB patients were scheduled to attend follow-up at the health facility weekly during the first 2 months of treatment and monthly in the next 6 months. During follow-up appointment day, each patient received health education from the CHW, received their TB medication and was reviewed clinically by the nurse in charge of TB at the facility. The CHWs and the nurses in charge would meet monthly to review their activities and to refresh their knowledge regarding TB. In the control groups, which were in the facilities that did not utilise CHWs; TB patients commencing treatment were advised on treatment schedule, treatment adherence and need for family support by the nurse at the facility. Unlike in the comparison group, these patients did not receive any additional support from a CHW.

### Variables

The primary outcome variable was TB Treatment adherence which was defined as; patients who were adherent in both treatment phases Intensive Phase (IP) and Continuous Phase (CP).Within the IP, attending to the scheduled follow-ups continuously to pick medication was the measure; a period equivalent to a minimum 49 continuous days to a maximum of 63 continuous days was considered adherent, while in the CP all patients with the following treatment outcomes; cured, treatment complete, died, and failure were adherent. Patients with outcomes of default and transfer out were non-adherent. The main exposure was utilization of CHWs. Other covariates that were used in this study included, demographics, type of TB, patient classification, acceptance of HIV screening, urban versus rural location.

### Data Analysis

STATA version 12 was used for statistical analysis. Descriptive statistics were used to explore the data and provide a description of the study subjects. Subjects were classified either as having utilized CHWs or not. These 2 groups were further grouped into adherent or non-adherent based on the study criteria. The association between baseline characteristics and adherence to TB treatment were evaluated with the chi square test and logistic regression. A stepwise selection procedure for variables was used to develop a multivariate model for assessing factors with different rates of adherence to TB treatment. These factors included age, gender, treatment setting, patient classification and type of TB. Tests of interaction were used to test whether the strength of associations varied according to setting (rural vs. urban) where CHWs were utilized and adherent to TB treatment. The qualitative data was typed into scripts and the information extracted from them was themed and interpreted by guided words, context, internal consistency, frequency and extensiveness of comments, specificity of comments and any big ideas.

### Ethics Statement

Purpose of this study and assurance of data confidentiality was explained to the Ministry of Health (MOH), the custodian of all patient data so as to seek approval for data retrieval from patient records. Written permission to use patient records was provided by the MOH and this was considered as an agreement to use patient records without their written consent because of their non-availability. The patient records received from the MOH was in the form of hard copies of treatment registers containing the full details of the patients including their names. The patient records were anonymized before data analysis by not including the names of the patients as part of data entry. Before any discussions were initiated for the FGDs and IDIs, verbal consent from participants was obtained. Written consent was not taken for these discussions to encourage the sharing out of ideas freely especially for participants who would be hesitant to talk about their opinions if their identification details were documented. The participants in the discussions were informed that all information collected would be treated as group contribution, while the in-depth interviews represented opinion from formal health workers who had engaged with CHWs. The participants were informed that the discussions would be written down by a research assistant and also audio recorded, to support the analysis of the information. The study protocol together with the permission to use patient records from the MOH and the use of informed verbal consent was approved by the scientific and ethical review committees of KEMRI before implementation of the study.

## Results

The study enrolled 2778 TB patients (Figure 1) and among them 1499 (54%) utilized CHWs for their TB treatment. The proportion of males and females were comparable in both the groups that utilized CHWs and that which did not (Table 1). Age group distribution was similar in the 2 groups with mean age and standard deviations falling within the same range. The urban setting in comparison with the rural setting contributed significantly to a higher proportion (70%) of patients utilising the CHWs (p<0.001). There was also a significantly higher proportion of pulmonary smear negative (PSN) patients (56%) utilizing CHWs for their treatment compared to pulmonary smear positive (PSP) 19% (p<0.001). A majority of the patients in this cohort were new TB patients (Table 1).

**Table 1:** Characteristics of Study Participants.

	CHWs utilized n = 1499*	CHWs not utilized n = 1279*	p- value
**Age**	29.88 (mean) SD 13.39	32.73 (mean) SD 14.24	<0.001
**Gender**			
Male	790 (52.70)	660 (51.60)	0.563
Female	709 (47.30)	619 (48.40)	
**Disease Classification**			
Pulmonary Smear Positive (PSP)	287 (19.15)	936 (73.18)	<0.001
Pulmonary Smear Negative (PSN)	839 (56.0)	40 (3.13)	
Extra Pulmonary TB (EPTB)	371 (24.75)	299 (23.38)	
Missing	2 (0.13)	4 (0.31)	
**Patient Classification**			
New	1128 (75.25)	1106 (86.47)	<0.001
Retreatment	329 (21.95)	110 (8.60)	
Missing	42 (2.80)	63 (4.93)	
**Accepted HIV Screening**			
No	87 (5.80)	287 (22.44)	<0.001
Yes	1412 (94.20)	992 (77.56)	
**Location**			
Rural	448 (29.89)	785 (61.38)	<0.001
Urban	1051 (70.11)	494 (38.62)	
**Adherence to treatment**			
No	215(14.34)	343 (26.82)	<0.001
Yes	1237 (82.52)	875 (68.41)	
missing	47 (3.14)	61 (4.77)	

*All data are n(%).

CHW, community health worker.

The overall treatment adherence of the cohort as a whole was 79%, on categorizing by use of CHWs, adherence among patients who had utilized CHWs was 83% compared to 68% among those that did not utilize CHWs (p<0.001). In comparison between the rural and urban set up adherence was 76% and 81.5% (p<0.001) respectively and when categorized by use of CHWs it was 73% and 90% (p<0.001) in the rural and urban set ups respectively.

Among the patients who were adherent, age group 25–34 years made up the highest proportions of those who were utilising CHWs (38% p = 0.015) and similarly contributed to the highest proportion in the group not utilising CHWs (33%, p = 0.803). The PSN patients were more likely to be adherent (56.5%, p<0.001) compared to the other disease classifications among the patients who had utilised CHWs. In the group that was not utilising CHWs, the PSP were more likely to be adherent (75%, p = 0.006). New TB patients utilising CHWs were significantly likely to be adherent (p = 0.032) than those not utilising CHWs. There was a higher proportion (94% p = 0.472) of the patients utilising CHWs who accepted HIV screening compared to those not utilising CHWs (79% p = 0.179). Location and utilization of CHWs combined did have an effect on treatment adherence as demonstrated in table 2. Among the patients who were adherent to TB treatment and utilising CHWs, 75% (p<0.001) were residing in the urban set-up compared to the 25% from the rural set up. Not utilising CHWs had higher adherence rates in the rural set up, 68% (p<0.001) compared to 32% in the urban set up.

**Table 2:** Treatment Adherence by potential risk factors, sorted by utilisation of CHWs.

	CHW Utilised n = 1237*		CHWs not utilised n = 875*	
	Adherent	p value	Adherent	p value
**Age**				
0–14	158 (12.77)	0.015	82 (9.37)	0.803
15–24	195(15.76)		126 (14.40)	
25–34	470 (38)		288 (32.91)	
35–44	275 (22.23)		202 (23.09)	
45–54	88 (7.11)		111 (12.69)	
55–64	34 (2.75)		40 (4.57)	
65+	14 (1.13)		20 (2.29)	
missing	3 (0.24)		6 (0.69)	
**Sex**				
Female	597 (48.26)	0.267	434 (49.60)	0.321
Male	640 (51.74)		441 (50.40)	
**Disease Classification**				
PSP	213(17.22)	<0.001	654 (74.74)	0.006
PSN	699 (56.51)		35 (4)	
EPTB	324 (26.19)		184 (21.03)	
Missing	1(0.08)		2 (0.23)	
**Patient Classification**				
New	938 (78.83)	0.032	749 (85.6)	0.115
Retreatment	263 (21.26)		87 (9.94)	
missing	36 (2.91)		39 (4.46)	
**Accepted HIV Screening**				
Yes	1162 (93.94)	0.472	691(78.97)	0.179
No	75 (6.06)		184 (21.03)	
**Location**				
Urban	928 (75.02)	<0.001	280 (32)	<0.001
Rural	309 (24.98)		595 (68)	

* All data are n(%).

CHW, Community Health Worker, PSP Pulmonary Smear Positive, PSN Pulmonary Smear Negative, EPTB Extrapulmonary TB.

Logistic regression was done on the data as a whole and this revealed that other co-variates did not have any additional influence on adherence other than the utilization of the CHWs as demonstrated in table 3 on the unadjusted arm. Utilisation of CHWs still remained a factor on its own as revealed by the adjusted odds ratios (table 3).

**Table 3:** Multivariate Regression Analysis of the Association between CHWs and TB Treatment Adherence.

	Unadjusted Adherence			Adjusted Adherence		
Exposure Variable	OR	95% CI	p value	OR	95% CI	p value
CHW	2.25	(1.86 –2.73)	<0.001	1.98	(1.51 –2.5)	<0.001
Age	1.00	(0.93–1.07)	0.886	1.00	(0.99 –1.00)	0.71
male	0.85	(0.70–1.02)	0.095	0.83	(0.68 –1.01)	0.07
Type of TB	1.28	(0.95–1.73)	0.097	1.19	(0.87 –1.63)	0.26
New vs Retreatment	1.21	(0.91–1.62)	0.180	1.26	(0.93 –1.69)	0.12
Accepted HIV Screening	1.20	(0.93–1.57)	0.154	1.20	(0.89– 1.60)	0.21
Location (urban/rural)	1.06	(0.87–1.30)	0.517	1.05	(0.88 –1.38)	0.38

To further demonstrate the effect of combining location and utilization of CHWs on treatment adherence, logistic regression analysis was done to find out the association. Table 4 shows a strong positive effect on combining the use of CHWs and location on treatment adherence, OR 8.02(95% CI 5.43–11.88, p<0.001).

**Table 4:** The Effect of combining location and use of CHWS on treatment Adherence.

Exposure Variable	OR	95% CI	p value
Location	0.45	(0.35 – 0.58)	<0.001
CHW	0.74	(0.56 – 0.97)	0.032
Location & CHW	8.02	(5.43 –11.88)	<0.000

CHW, Community Health Worker.

Utilisation of CHWs was most effective in the urban set-up, OR 2.65 (95% 2.02–3.48, p<0.001) in comparison to the rural set up as indicated in table 5.

**Table 5:** The Effect of location on treatment adherence.

Exposure Variable	OR	95% CI	p value
Urban & no CHWs	0.45	(0.35–0.58)	<0.001
Rural & CHWs	0.74	(0.56–0.97)	0.032
Urban & CHW	2.65	(2.02–3.48)	<0.001
Rural & no CHWs	ref		

CHW, Community Health Worker.

### Results from qualitative data

A majority of the CHWs who participated in the FGDs were between the ages of 25–30 yrs and at least 75% were female. They all had received the 3 day basic training on their roles as CHWs for TB care. They were all resident in the communities they served and spoke the same language as the community. From the discussions a description of their work included; linking people to health care, providing informal counselling, support and follow-up, making home visits, documenting their activities. They were all literate.

Some factors perceived to influence CHWs' performance were mentioned as: community support and confidence, the continued training and the monthly meetings with their supervisors combined with the cooperation from formal health workers. The availability of logistics to facilitate their work was reported as an important factor in sustaining them to continue working. This included a monthly allowance of USD 27 and a fee for tracing defaulters of USD 1.4. In the urban set-up the attrition rate among the CHWs over the 5 year period was about 16% compared to the rural set-up of 20%. One major reason that was mentioned as the reason for resigning from doing the work despite the continuous availability of support was the opportunity of getting a better source of income.

Factors that may have limited the performance of their work were mentioned and included, coverage of long distances for home visits resulting in lesser visits monthly. This mainly affected the rural site which reported that on average 1 CHW could only manage 20 visits/month while the urban site, a CHW was able to do 50 home visits/month on average. Other limiting factors mainly from the rural site included stigma towards TB from the community and cultural beliefs against the conventional treatment of TB, so that alternative treatments such as traditional medicine were options for use. In the urban site missing clients at home and sometimes insecurity threats were mentioned as factors that could limit their performance.

Two formal health workers involved in the supervision of the CHWs provided information on the utilization of CHWs through an in-depth interview. They reported that they recognized the importance of the CHWs complimenting their work. They felt the good treatment adherence rates would never have been achieved without the support of the CHWs. In both the rural and urban set up, the recruitment of the CHWs involved the community health committees that were composed of both lay leaders from the community and health workers from the facility.

## Discussion

This study showed that treatment adherence among TB patients who had utilized CHWs was higher (83%) than among those that did not utilize CHWs 68% (p<0.001). These findings are similar to those of previous studies [35], [36], [37] that demonstrated use of CHWs can effect good outcomes of treatment. Various factors have been attributed to the positive influence among patients on TB treatment including; the attitude of both the health facility personnel and the community [38]. In both urban and rural settings the CHW programmes were supported by health development partners supporting the Ministry of Health. This programme involved well defined tasks for the CHW; there was provision of capacity building through DOT training and regular refresher training, support supervision and a rewarding and recognition system for tasks done. The health workers in the facility recognized the importance of the CHWs complimenting their work as was revealed in their in-depth discussions. They felt the good treatment adherence rates would never have been achieved without the support of the CHWs. In both the rural and urban set up the recruitment of the health workers involved the community health committees that were composed of both lay leaders from the community and health workers from the facility. The involvement of this committee indicated community acceptance of the tasks of the CHWs and its positive attitude towards the health services provided by the formal health services. On average the CHWs in the urban and the rural set up had continuously worked for the community for the 5 years the study analysed. The sustainability of the CHWs may be most attributed to the continuous motivating factors that were mentioned by the CHWs as reasons that encourage them to support TB treatment adherence. This included the continuous support supervision, regular trainings, provision of incentives and recognition of work done well. These findings concur with other studies [38], [39].

The treatment adherence rate demonstrated in this study of 83% when CHWs are utilised is much lower than that which has been shown by other studies [40], [41]. These 2 studies revealed that if adherence is measured by testing Isoniazid in the urine of the patients at one point in time during their treatment, the rates of adherence were 97.6% and 95.7% respectively. While if adherence was measured also at one point in time by using patient reports the adherence rate was 95.2% [40] and if measured by visual analogue scale (VAS) within this same study the rate was 95.2%. The inconsistence observed is explained by the different methodologies used to determine adherence in this study and the other studies. It may also not be suitable to compare these adherence rates with those of this study, because this study assessed the rate of adherence throughout the full course of TB treatment and not at one point during treatment as the other studies did.

This study showed that utilising CHWs for TB treatment adherence was most effective in the urban set up compared to the rural set up OR 2.65 (95% 2.02–3.48, p<0.001). This is a similar finding from an evaluation study done by Dick et al. which revealed that using CHWs in the rural set up did not significantly improve treatment adherence [37]. Another study done in Brazil revealed similar findings, that utilising CHWs in certain urban settings enhanced treatment adherence [42]. The differences between the rural and urban set ups may be explained by the differences that were described through the discussions in this study from the CHWs and the health workers. In the urban, the CHWs were able to do more home visits compared to the rural because the distances were shorter and the terrain manageable. Though the CHWs in both sites performed the same duties the local and cultural setting varied, possibly the socioeconomic status and cultural beliefs in the rural area may have limited full access to quality health care. In the urban set up communication between clients and CHWs or between CHWs and the formal health care worker was much easier compared to the rural set-up especially with the use of cell telephones.

This study had various strengths. It used standard tools (patient treatment records) to define treatment adherence and the analytic procedures applied, allowed control of various confounding factors. The use of findings from the FGDs and IDIs to explain some of the differences found in the urban and the rural set up is an additional strength to this study. Using programmatic data by this study strongly supports the intervention of utilising CHWs in the management of TB treatment. The positive effect of utilising CHWs in a programmatic setting as found in this study can be of value for areas outside the context of TB especially for the management of chronic illnesses.

Only four clinics were included in the study there may have been a possibility of unique features of the clinics having influenced the study results. This limitation may result in the application of these findings to only similar settings. Another limitation of the study was that the study was not able to get information on other factors that may influence treatment adherence including level of education, occupation, travel distance and socio-economic status.

### Conclusions

Utilisation of CHWs in the treatment of TB resulted in better TB treatment adherence compared to not utilising CHWs. The results of this study revealed that other clinical or demographic factors such as age and gender of the patients did not influence the effects of utilising the CHWs. The use of data from a programmatic setting lends support for the utilization of CHWs in the management of TB and possibly other chronic illnesses.

Utilisation of CHWs in the management of TB in an informal settlement within an urban setting resulted in a high rate of TB treatment adherence compared to the rural setting. In planning for the utilization of CHWs in the rural set up many of the factors that may limit the performance of the CHWs need to have strategies that reduce their influence against the effects of utilising CHWs.
